# The Impact of Coping Strategies and Perceived Family Support on Depressive and Anxious Symptomatology During the Coronavirus Pandemic (COVID-19) Lockdown

**DOI:** 10.3389/fpsyt.2020.587724

**Published:** 2020-11-13

**Authors:** Rachele Mariani, Alessia Renzi, Michela Di Trani, Guido Trabucchi, Kerri Danskin, Renata Tambelli

**Affiliations:** ^1^Department of Dynamic and Clinical Psychology, Sapienza University of Rome, Rome, Italy; ^2^Department of Mental Health, ASL RM6, Albano Laziale, Italy; ^3^Princeton Psychological Services, Princeton, NJ, United States

**Keywords:** COVID-19, coronavirus pandemic, depression, anxiety, coping strategies, perceived support

## Abstract

The coronavirus pandemic represents a severe global crisis, affecting physical, and psychological health. Lockdown rules imposed to counteract the rapid growth of COVID-19, mainly social restrictions, have represented a risk factor for developing depressive and anxious symptoms. The research aims are to explore the effect of coping strategies and perceived social support on depressive and anxious symptomatology during the COVID-19 pandemic. Ninety-six healthy people (46 males, mean age = 39.3; *SD* = 16.6) completed through on-line platform: Socio-demographic questionnaire, Coping Inventory for Stressful Situations (CISS), Multidimensional Scale of Perceived Social Support (MSPSS) and Symptom Checklist-90-Revised (SCL-90-R), 3 weeks after the imposition of lockdown restrictions. SCL-90-R Depression scores showed significant positive correlation with CISS Emotion (*r* = 0.85; *p* = 0.001) and Avoidant (*r* = 0.34; *p* = 0.018), a significant negative correlation with MSPSS Family support (*r* = −0.43; *p* = 0.003). SCL-90-R Anxiety scores showed a significant positive correlation with CISS Emotion (*r* = 0.72; *p* = 0.001) and Avoidant (*r* = 0.35; *p* = 0.016). No significant correlations between both CISS Emotion and Avoidant scales with social support emerged. Two Multiple Linear Regression analysis were performed using, respectively, SCL-90-R Depression and Anxiety scores as dependent variables, and the CISS and MSPSS scales, age, and gender as predictors. The first regression model (*R*^2^ = 0.78; adjusted *R*^2^ = 0.75) revealed CISS Emotion (β = 0.83; *p* = 0.001) and MSPSS Family support (β = −0.24; *p* = 0.004) had a predictive effect on SCL-90-R Depression scores. The second regression model (*R*^2^ = 0.52; adjusted *R*^2^ = 0.472) revealed that only CISS Emotion (β = 0.71; *p* = 0.001) predicted the SCL-90-R Anxiety scores. In conclusion, during the COVID-19 pandemic lockdowns, coping focus on emotions seemed to increase anxious and depressive symptoms, probably due to the uncontrollable nature of the stressful event and the high emotional response. Family support which reduces the sense of loneliness had an exclusive role in mitigating depressive symptoms. These results highlight the importance of promoting psychological strategies to improve emotional regulation skills, reducing isolation from family, to prevent mood symptomatology in healthy citizens during large-scale health crises.

## Introduction

As nations around the globe continue their battle with the COVID-19 pandemic, it has become clear that people in some regions will experience repeated lockdown or quarantine periods. If this unfortunate reality is to be faced and endured, it is important that mental health providers are armed with accurate information about how to help the public survive these periods of isolation and inactivity with minimal psychological impact. When lockdown procedures began, protection of physical health was the top priority, but those familiar with the impact of phenomena like isolation, loneliness, and unemployment on mental health braced for an additional threat.

Now that initial lockdown limitations all around the world are either beginning to lift, or becoming the “new normal” in places where they have continued for many months, some research is emerging that will assist in the development of environmental and psychological interventions to lessen their impact moving forward. The authors of this paper hope with the present research to make a contribution to that work, specifically in the areas of coping strategies and social support. Clinicians who treat individuals with symptoms of depression and anxiety know that a healthy social and physical environment is critical for maintenance of balanced mental health. Indeed, the first clinical recommendations for many of the patients presenting with such symptoms, particularly those with depression, are often to increase social interaction and support, engage in a wider variety of activities outside the home, and engage in activities that foster a sense of mastery, including work. With these options severely limited due to lockdowns, it was clear that many people would find it difficult to navigate their mood, anxiety, and other mental health challenges. Studies carried out in previous instances of highly infectious diseases and pandemics have shown that social isolation produces serious psychological and emotional repercussions (1, 2). Taylor et al. (3) found that 34% of quarantined horse owners reported psychological distress during the equine influenza epidemic compared to 12% in the general population. In another study, parents who experienced a variety of disease containment lockdowns were found to endorse 6% more trauma-related psychological symptoms than parents who had not experienced lockdowns (4).

Research that has been conducted thus far in countries impacted by COVID-19 supports the hypothesis that the pandemic and related lockdowns have had a significant impact on mental and physical health (5). Some of this research has focused on stress and trauma symptomatology. Relatively significant correlations have been found between COVID-19- PCL-5 [a version of the Posttraumatic Stress Disorder Checklist for DSM-5 developed by (6), modified by (7)] scores, general distress and sleep disturbances. A high percentage of PTSD symptoms (29.5%) was found in the Italian population (7). Survey findings from Liu et al. (8), indicated that the prevalence of post-traumatic stress symptoms in hard-hit areas of China ~1 month after the emergence of the virus was 7%, and had particularly impacted women. In a Spanish study (9) it was reported that 41% of their survey respondents reported feeling stressed. Wang et al. (10) reported that 8.1% of their Chinese respondents were experiencing moderate to severe stress. The COVID-19 pandemic is likely to be considered a traumatic event, especially by those whose life circumstances have been affected. The pandemic has also presented particular challenges for individuals who struggle with substance use due to the fact that social isolation and despair are risk factors for the development and exacerbation of addiction (11, 12). Professionals across multiple disciplines have raised alarms about the potential for increased family violence or intimate partner violence [e.g. (13–15)] as a result of increased exposure to exploitative relationships and economic stress, as well as reduced support.

The social and economic features of the pandemic have created conditions that are strongly associated with mental health issues. Information about the pandemic has changed regularly, as epidemiologists and other professionals have tracked its progress and examined its characteristics, which has meant that the everyday citizen may feel that they are lacking in knowledge or distrustful of the latest findings. Individuals in affected areas have worried about how they will meet basic needs like food and medicine, and have struggled with fear of contagion. Lockdown rules have reduced contact with social and professional connections while producing concerns about financial stability.

Brookings Institution (16) reported thirty-eight million people in 20 wealthy democracies around the world had filed for unemployment insurance over the course of the pandemic. Prior research on the psychological impact of unemployment has been quite clear; a meta-analysis (17) of 324 cross-sectional and longitudinal studies found that on average, 34% of unemployed people experienced psychological problems, compared with 16% among those who were employed. Burnout is one additional social factor that may become increasingly relevant to the development of pandemic-related psychopathology as pressures on families—especially parents—continue to mount; it may resonate deeply with those engaged in intense, concurrent domestic and professional labor during this crisis, and may produce some of the same psychopathology (18–20). Burnout in individuals in the health profession in particular should be taken into consideration as a social implication of the pandemic (21).

Anxiety and depression symptoms related to COVID-19 pandemic have been a topic of intense interest among researchers. Their proliferation among the populations of many of the affected nations appears to be widespread according to early studies. Wang et al. (10) reported that 16.5% of their respondents in China reported moderate to severe depressive symptoms and 28.8% reported moderate to severe anxiety symptoms. Cao et al.'s (22) survey of Chinese undergraduate college students found that 0.9% of the respondents were experiencing severe anxiety, 2.7% moderate anxiety, and 21.3% mild anxiety. Similarly, Huang and Zhao (23) found significant psychopathology among their participants in China−35.1% endorsed symptoms of generalized anxiety and 20.1% endorsed depressive symptoms. Similar findings have been reported in Nepal [(24); a preprint study indicating depression, anxiety and depression-anxiety co-morbidity reported by 34, 31, and 23.2%, respectively], the Philippines [(25); COVID stress significantly predicted depression and anxiety], and India [(26); depression, anxiety and insomnia symptoms reported by 12.7, 9, and 21%, respectively]. In Europe, Rodriguez-Rey et al.'s (9) study found that 25% of their Spanish respondents showed mild to severe levels of anxiety and 41% reported depressive symptoms. In Bäuerle et al.'s (27) study, on 15.704 German participants, the overall prevalence of elevated anxiety and depressive symptoms was 44.9 and 14.3%, respectively. Solomou and Constantinidou's (28) study in Cyprus was roughly equivalent in its findings-−41% of respondents reported symptoms of mild anxiety; 23.1% reported moderate-severe anxiety symptoms; and 48% reported mild and 9.2% moderate-severe depressive symptoms. As specifically regards Italy, Gualano et al. (29) found that during the last 14 days of lockdown on 1,515 participants enrolled in their national survey the prevalence of depression and anxiety symptoms was 24.7 and 23.2%, respectively. In their study, increasing age, an absence of work-related troubles and being married or being a cohabitant reduced the likelihood of at least one mental health outcome. Furthermore, in a recent meta-analysis the prevalence of anxiety symptomatology, investigated in 17 studies was obtained as 31.9%, whereas the prevalence of depressive symptoms, investigated in 14 studies, was reported as 33.7% (30). While the above studies focused mainly on the general population, the currently available research suggests that frontline healthcare workers in particular have experienced increased depression and anxiety symptoms [see (31) for an overview of ten studies conducted in Asia on this topic]. Similarly, Cao et al. (22) found that during the COVID-19 lockdown their sample of college students experienced economic effects, and effects on daily life, as well as delays in academic activities, that were positively correlated with anxiety symptoms. Anxiety and depression symptoms may be appropriate reactions to these extreme circumstances, but over time or with increased intensity, they may become maladaptive and impair functioning (32).

Recent research has explored the effect of social support and coping strategies in relation to anxiety and depression. During the pandemic, greater levels of perceived social support appears to have been serving as a protective factor for affected individuals (22). One recent study shows that different levels of social support for medical staff were significantly correlated with self-efficacy and sleep quality and negatively correlated with the degree of anxiety and stress (33). However, social support is a multidimensional factor and loneliness can not necessarily be identified and assessed based on the number or absence of social contacts. Both depend on an individual's self-perception of “how I feel supported or alone.” Bruwer et al. (34) asserted that social support is a complex and multidimensional construct whose explanation is still the subject of numerous interpretations. Thoits (35) suggested that social support operates primarily as “coping assistance” with the negative effects of stress, which increases self-esteem and a sense of control over the environment. Social support is in contrast with loneliness, which may lurk in the hearts of people who are ostensibly surrounded by and engaged with others. Often it is preceded by significant changes in the person's life. It has a strong negative impact on mental and physical health, including premature death at rates comparable to obesity and smoking (36).

During the COVID-19 pandemic, it is very important to evaluate the difference between the effect caused by the physical distancing imposed by lockdowns and the extent to which individuals subjectively feel lonely or feel supported by others. Social isolation is evident to an observer as a state in which a person is neither in close proximity with nor are they interacting with others. They may not actually feel alone. It is also important to remember that social support is complex and can vary based on the type of support provided by significantly different figures, such as family, friends or others (37). Research has also highlighted that different kinds of support can have different impacts on stress reduction (38); in fact, support specifically from family and friends during the COVID-19 pandemic appears to have been helping people feel sustained and share their feelings (39). In addition to social support, various coping strategies appear to have differing effects in preventing or fostering psychological symptoms. Extant literature regarding the combined psychological responses and coping methods used by the general population in past outbreaks has shown that coping strategies have included problem-focused coping (seeking alternatives, self- and other-preservation) and seeking social support to mitigate anxiety and depression (40). Given the nature of the COVID-19 pandemic, coping strategies have been affected by announcements of clear rules for citizens to follow; social media communications and expert advice encouraged a task-oriented coping strategy. These factors have helped people to try to behave calmly and appropriately (41). This complements previous research which has demonstrated that high levels of emotion-oriented coping and low levels of task-oriented coping tend to correlate positively with burnout in healthcare workers (42, 43). As specifically regards the coping behaviors associated with decreased anxiety and depressive symptoms during the COVID-19 pandemic and lockdown in a Spanish study (44) it has been found that following a healthy/balanced diet and not reading news/updates about COVID-19 very often were the best predictors of lower levels of anxiety symptoms, whereas following a healthy/balanced diet, and a daily routine, not reading news/updates about COVID-19 very often, taking the opportunity to pursue hobbies, and staying outdoors or looking outside were the best predictors of lower levels of depressive symptoms. Furthermore, in a Swiss study (45) the coping strategies associated with reduced emotional distress in young adults included keeping a daily routine, physical activity, and positive reappraisal/reframing.

Given the proliferation of depression and anxiety symptoms as primary psychological consequences of the pandemic and lockdown procedures, it is increasingly clear that interventions must be developed quickly to soften the impact of additional lockdowns and the ongoing threat of the virus, as well as potential future pandemics. With that in mind, the present authors have explored how various coping strategies have increased or decreased these symptoms, and worked to discover the role of social support in this process. The research aims are to explore specific how coping strategies and perceived social support have been impacting depressive and anxious symptomatology during an extended period of lockdown rules during the COVID-19 pandemic in a healthy sample. In particular it is hypothesized that:

- The adoption of task-focused coping strategies is related to less anxiety and depressive symptoms;
- Emotional coping is related to increment of anxiety and depression symptoms;
- Family support in particular is related to fewer symptoms of anxiety and depression during the COVID-19 pandemic.

## Materials and Methods

### Participants

The study focused on healthy Italian individuals enrolled based on the following inclusion criteria:

- having been subject to lockdown social restrictions rules;
- between the ages of 18 and 70
- having adequate understanding of the Italian language and living in Italy at the time of the lockdown;
- possessing the technical ability to access to the on-line platform to complete questionnaires.

We excluded people who had previously received a psychiatric diagnosis; those who take medication for psychiatric reasons; and individuals who were working as healthcare professionals during the pandemic. A total of 98 healthy subjects (46 males) participated in the study. The participants had a mean age of 39.3 (*SD* = 16.6), Additionally, 45.8% reported an educational level of 13 years, 41.7% of 16 years, and 12.5% over 16 years. Similarly, 45.8% indicated that they were married/cohabiting; 25.2% were unmarried/not cohabiting and living independently (may have had roommates); 8.3% were divorced; and 20.7% were single living with their families of origin.

### Procedure

The survey protocol received the ethical approval by the Sapienza University Ethics Committee. The study was conducted in the first week of April, 2020, 3 weeks after the imposition of lockdown restrictions. The choice of performing the evaluation 3 weeks after the imposition of lockdown restrictions depended on the need to let some time to pass from the imposition of restrictions in order to be able to evaluate their effects after a first period of new of the event. This since the aim was not to investigate population's immediate reaction to lockdown restrictions but the impact of this prolonged difficult situation on people's psychological health. The participants were invited to complete and on-line survey asking them to share their insights into how people feel about the global health emergency and how they are coping with it. The participants were enrolled using snowball sampling. The surveys were made available through an on-line platform where participants gave their informed consent before completing the self-administered questionnaire.

### Measures

#### Socio-Demographic Questionnaire

A socio-demographic questionnaire was designed to collect information concerning age, gender, education level, social status and occupation.

#### The Symptom Checklist-90-Revised

The Symptom Checklist-90-Revised [SCL-90-R; (46)] is a 90-item self-report inventory which measures psychological and psychosomatic symptoms occurring in psychiatric, medical, and general population participants. Each item is a description of a psycho-physical symptom and is rated by respondents on a five-point Likert scale (0–4) from having caused no discomfort to extreme discomfort during the past week. The SCL-90-R has 9 subscales: (1) Somatization, (2) Obsessive-Compulsive, (3) Interpersonal Sensitivity, (4) Depression, (5) Anxiety, (6) Hostility, (7) Phobic Anxiety, (8) Paranoid Ideation and (9) Psychoticism. The sum of all 9 subscales is the Global Severity Index (GSI), which can be used as a summary of the test, reflecting overall psycho-physical distress. In the present study the focus was placed on the Depression and Anxiety scale scores (47). The SCL-90-R showed adequate test–retest reliability, internal consistency and concurrent and discriminant validity. Cronbach's alpha of subscales in the present study ranged from 0.76 to 0.87.

#### Coping Inventory for Stressful Situations

The Coping Inventory for Stressful Situations [CISS; (48, 49)] is a questionnaire of 48 items measured on a Likert scale from 1 (not at all) to 5 (very much). Respondents are asked to indicate how much they engage in these types of activities when they encounter a difficult, stressful, or upsetting situation. The questionnaire measures along three coping dimensions: (1) Task-oriented coping, in which the main emphasis is placed on tasks or planning, and on attempts to solve problems; (2) Emotion-oriented coping, in which individuals engage in emotional reactions that are self-oriented. It includes emotional responses such as getting angry, becoming tense, as well as self-preoccupation and fantasizing, as in daydreaming reactions. (3) Avoidance-oriented coping describes activities and cognitive changes aimed at avoiding the stressful situation. The test showed good psychometric properties including internal-consistency, test-retest reliability, and concurrent and discriminant validity (50).

#### Multidimensional Scale of Perceived Social Support

The Multidimensional Scale of Perceived Social Support [MSPSS; (51)] is a self-report measure of subjectively perceived social support. The questionnaire is composed of 12 items rated by respondents on a Likert scale ranging from 1 (totally false for me) to 7 (totally true for me). The questionnaire measures three different sources of support: Family (4 items), Friends (4 items), and Significant Other (4 items), and there is also a total support score (12 items). The questionnaire demonstrated good internal and test-retest reliability (52).

### Data Analysis

All statistical analyses were performed using the Statistical Package for Social Science version 25 (SPSS version 25) for Windows (IBM, Armonk, NY, USA). Data were reported as frequencies and percentages for discrete variables, and as means and standard deviations for continuous variables. Pearson's correlation analysis was used to measure the association between depression/anxiety levels, coping strategies, perceived social support, age and gender. Two Multiple Linear Regression models were performed using, respectively, Depression and Anxiety scores as dependent variables, and age, gender, CISS and MSPSS dimensions that were significant from the correlation analysis as predictors. A *p* < 0.05 was considered significant.

## Results

In the sample that was evaluated, 33.3% of the participants showed elevated symptoms of depression and 35.4% elevated symptoms of anxiety (in both cases, scores equal to or >1 in the Depression and Anxiety SCL-90-R scores).

The questionnaire mean scale scores of the participants are presented in Table 1.

**Table 1:** Participants' questionnaire means scale scores.

**Variables**	**M**	**Sd**
**SCL-90-R**		
Depression	0.86	0.66
Anxiety	0.78	0.53
Global Severity Index	0.62	0.46
**CISS**		
Emotion	35.68	12.49
Avoidant	45.38	8.12
Task	56.34	9.43
**MSPSS**		
Family	5.25	1.03
Friends	5.45	1.22
Significant others	4.06	0.88
Total	5.48	0.85

*SCL-90-R, Symptom Checklist-90-Revised; CISS, Coping Inventory for Stressful Situations; MSPSS, Multidimensional Scale of Perceived Social Support*.

In regards to the correlational analysis (see Table 2), SCL-90-R Depression scores showed a significant positive correlation with CISS Emotion (*r* = 0.84; *p* = 0.001) and CISS Avoidant (*r* = 0.34; *p* = 0.018) coping styles, and a significant negative association with MSPSS Family support (*r* = −0.426; *p* = 0.003). SCL-90-R Anxiety scores showed a significant positive correlation with CISS Emotion (*r* = 0.719; *p* = 0.001) and CISS Avoidant (*r* = 0.35; *p* = 0.016) coping styles. No significant associations between either CISS Emotion or Avoidant scales with social support emerged.

**Table 2:** Correlation between depression and anxiety, with coping, and social support dimensions.

	**SCL-90 R Depression**	**SCL-90 RAnxiety**	**CISS Emotion**	**CISS Avoidant**	**CISS Task**
Age	−0.232	−0.170	-	-	-
Gender	0.194	0.194	-	-	-
CISS Emotion	0.849^**^	0.719^**^	-	-	-
CISS Avoidant	0.343^*^	0.349^*^	-	-	-
CISS Task	−0.272	−0.173	-	-	-
MSPSS Family	−0.426^**^	−0.277	−0.238	−0.007	0.188
MSPSS Friends	0.130	0.190	0.208	0.259	0.040
MSPSS Significant others	−0.208	−0.118	−0.266	−0.106	0.120
MSPSS Total	−0.182	−0.064	0.021	0.178	0.126

** *p < 0.01*;

* *p < 0.05*.

*SCL-90-R, Symptom Checklist-90-Revised; CISS, Coping Inventory for Stressful Situations; MSPSS, Multidimensional Scale of Perceived Social Support*.

A first multiple linear regression model was performed using the SCL-90-R Depression score as dependent variable and age, gender, CISS Emotion and Avoidant and MSPSS Family support (significant in the correlational analysis) as independent variables. The model explains the 78% of the Depression scores (*R*^2^ = 0.78; adjusted *R*^2^ = 0.75), thus indicating an adequate fit of the model tested. The independent variables that showed a significant effect were: CISS Emotion (β = 0.83; *p* = 0.001) and MSPSS Family support (β = −0.24; *p* = 0.004).

A second multiple linear regression model was performed using the SCL-90-R Anxiety score as the dependent variable and age, gender, CISS Emotion and Avoidant (significant in the correlational analysis) as independent variables. The model explains the 51% of the Anxiety scores (*R*^2^ = 0.52; adjusted *R*^2^ = 0.47), thus indicating an adequate fit of the model tested. The only independent variable that showed a significant effect was CISS Emotion (β = 0.71; *p* = 0.001).

## Discussion

During the COVID-19 pandemic, social distancing has been implemented in many countries, including Italy, to interrupt viral transmission and delay the spread of infection. These measures have come at a cost of socially isolating many people, putting their mental health at risk, since social isolation can lead to loneliness, a subjective psychological state identified through introspection that has been found consistently to be associated with depression, suicidal ideation and anxiety (53–59).

Coping styles and the perceived social support both appear to contribute to individuals' management of the stress of social isolation and the sense of loneliness that can derive from it.

The present work therefore aims to evaluate the relationships among specific coping strategies, perceived social support and anxious/depressive symptoms in the Italian general population exposed to COVID-19 during the lockdown period.

Consistent with previous studies [e.g., (9, 10, 22–24)], the presence of depression and anxiety symptoms was found in the sample we examined; specifically, 33.3% of the participants showed elevated symptoms of depression and 35.4% had elevated symptoms of anxiety (in both cases, scores equal to or <1, in the Depression and Anxiety SCL-90-R scores). These percentages appear, from a qualitative point of view, comparable or, in some cases, higher than those found in the other studies [depressive symptoms: 16.5% in (10); 20% in (23); 12% in (26); 9.2% in (28); anxious symptoms: 25% in (22); 9% in (26); 25% in (9)]. It seems important to consider that Italy was one of the first countries to be significantly affected by COVID-19 and that it immediately instituted complete social isolation measures. The speed with which the phenomenon had spread in some regions of the country, the lack of knowledge relating to the management of the virus and the uncontrollability of the pandemic all contributed to the stressful experience that may have led to an intense and not-regulable emotional reaction, expressed in anxiety and depressive symptoms.

Regarding the relationships among coping styles, perceived social support and depressive/anxious symptoms, correlational analysis showed that depressive symptoms were positively correlated with emotional coping style, avoidant coping style and low social support, specifically related to family support. The hypothesis that using a task-oriented coping style would be protective was not supported, but a relationship between the use of the emotional reaction as a strategy to cope with the stressful event and the presence of depressive symptoms was found, as hypothesized. It is possible that, in the face of such an uncontrollable, generalized, new and indefinite event as the COVID-19 pandemic, the emotional reaction can be very intense. Attempting to use it as a strategy to manage the condition, external and internal, can therefore be not only inappropriate but also frustrating, and increase stress levels. The lockdown rules, also, restricted people to their homes, a situation which may have threatened their sense of efficacy as their freedom to solve problems and create strategies was limited. Other research has demonstrated that this phenomenon may have been different for nurses, who engaged in more task-oriented coping strategies (60). In a condition of high dysregulation, even the attempt to focus on the concrete problem management can fail, especially when the problem is unknown and not controllable. In these cases, it may be more effective to avoid focusing on emotions, since doing so can lead to depressive symptoms, perhaps because the uncertainty inherent in the situation makes it impossible to fully process them.

Moreover, the lack of a source of regulation, such as the presence of significant relationships, can exacerbate emotional dysregulation, increasing loneliness and depressive symptoms. The specific aspect of social support linked to depression in this study was perceived family support. This aspect appears important because in Italy people have been forced to stay at home for more than 2 months, and therefore they have lived, in most cases, only with family members. Even in the Phase II of pandemic management following full lockdowns (Phase II started in Italy 3rd June after Phase I, which was characterized by total lockdown. Phase II was a Government strategy to maintain social distance but re-open all work activities), people were granted the freedom to visit only relatives, but not friends. Family relationships have taken on an important role, acting as a buffer against stress if they were adequate and supportive, or as a risk factor for depression, if perceived as deficient and inadequate. Loneliness refers to subjective dissatisfaction with the discrepancy between the perception of one's desired social network and that which is apparent to the individual (61, 62). It is not necessarily about being alone, but is connected to the perception of being alone and isolated that matters most. In other words, it is a state of mind which affects one's ability to find meaning in their life and creates unpleasant feelings of deficiency in social relations. It is important to highlight the distinction between social isolation and loneliness. What we observed in this study was not a depressive phenomenon linked to social isolation, but rather a sense of loneliness related to the perception that family relationships—the only sources of support at the time of the lockdown—were unable to perform this function. Regression analysis confirmed the specific role of the emotion oriented coping style and family support as predictors of depressive symptoms, supporting the possibility that an avoidant strategy was secondary to the failure of the emotion-oriented coping style.

Regarding anxious symptoms, correlation analysis showed a relationship between anxiety and emotion oriented and avoidant coping styles, whereas the regression analysis confirmed only the role of the emotional strategy in predicting anxious symptoms. Analysis did not demonstrate a relationship between perceived social support and anxiety. Depression and anxiety can be considered to be different symptomatic expressions of the same state of emotional dysregulation, which in one case results in a chaotic expression of the emotion and, in the other, in an emotional flattening. Even in the case of anxiety, too intense emotions cannot be used effectively to manage stress, as they need to be identified and regulated first. Social support seems to be specifically related to depression, probably mediated by the sense of loneliness.

The results of this study can be useful to orient not only psychological interventions for the general population in the post-emergency period, but also to direct health policies that take into account the psychological health of citizens. In the first area, implementing health promotion interventions aimed to strengthen emotion management strategies in stressful conditions could be useful; as regards health policies, it could be useful to consider the possibility of supporting significant social relationships (not just family ones) as much as possible through policies of improvement of virtual spaces where people can gather. The greater use of the internet and social media that the pandemic has engendered could be the basis for the construction of online support interventions for individuals and for small groups. Many psychological services and research projects are moving toward promoting teletherapy and other treatments that can be provided remotely, mostly in order to address the needs of the general public, but also to support medical professionals who have suffered enormous stress providing treatment during the pandemic (63).

There are several limitations to the present study. First is the limited size of the sample, which cannot be representative of the full Italian population, and so certainly cannot necessarily represent all populations globally. It should also be noted that in a lockdown period people may not be willing to describe their internal states without the supportive function that the relationship with a clinician can offer. In fact, in order for self-report measures to have good validity, the individuals completing them must be able to accurately assess their internal states; this can limit their utility, especially in clinical populations (64). In future research, utilizing a clinical interview would provide a more accurate assessment of participants' health status.

In addition, the use of the web for data collection, while allowing for contact with the general public during a lockdown period, does limit the findings to the population of individuals who voluntarily participated and does not allow analysis of those who chose not to participate. Moreover, the cross-sectional nature of the study does not allow us to identify cause-and-effect relationships, and for this reason the authors hope to be able to collect data on the psychological health of the sample observed with a follow-up of 6 and 12 months from the first sampling.

The present study presents also some strengths, as the importance of the topic investigated that is aimed to increase the knowledge regarding the impact of both the COVID-19 pandemic and the lockdown rules on psychological health. A further strength is the focus given to the exploration of the association between the perception of social support and depressive and anxious symptomatology, as regards the clinical and therapeutic relevance of these findings.

## Data Availability Statement

The raw data supporting the conclusions of this article will be made available by the authors, without undue reservation.

## Ethics Statement

The studies involving human participants were reviewed and approved by Ethics committee of Department of Dynamic and Clinical Psychology, University of Rome, Sapienza. The patients/participants provided their written informed consent to participate in this study.

## Author Contributions

RM contributed to all the phases of the study from conception and design of the study, results interpretation, and writing manuscript. AR performed the statistical analysis, contributed to results interpretation, and in writing the manuscript. MD contributed in data analysis, in results interpretation, and in writing the manuscript. GT contributed to conception and design of the study, organized the database. KD contributed in writing and editing of the manuscript. RT contributed to the interpretation of the results and supervision of the work. All authors contributed to manuscript revision, read, and approved the submitted version.

## Conflict of Interest

The authors declare that the research was conducted in the absence of any commercial or financial relationships that could be construed as a potential conflict of interest.

## References

[1] Hawryluck L Gold WL Robinson S Pogorski S Galea S Styra R SARS control and psychological effects of quarantine, Toronto, Canada. Emerg Infect Dis. (2004) 10:1206–12. 10.3201/eid1007.030703 15324539 PMC3323345

[2] Brooks SK Webster RK Smith LE Woodland L Wessley S Greenberg N. The psychological impact of quarantine and how to reduce it: rapid review of the evidence. Lancet. (2020) 395:912–20. 10.1016/S0140-6736(20)30460-8 32112714 PMC7158942

[3] Taylor MR Agho KE Stevens GJ Raphael B. Factors influencing psychological distress during a disease epidemic: data from Australia's first outbreak of equine influenza. BMC Public Health. (2008) 8:347. 10.1186/1471-2458-8-347 18831770 PMC2571100

[4] Sprang G Silman M. Posttraumatic stress disorder in parents and youth after health-related disasters. Disaster Med Public Health Prep. (2013) 7:105–10. 10.1017/dmp.2013.22 24618142

[5] Mazza M Marano G Antonazzo B Cavaretta E Di Nicola M Janiri L. What about heart and mind in the covid-19 era? Minerva Cardioangiol. 32397693 10.23736/S2724-5683.20.05309-8

[6] (2020). Blevins CA Weathers FW Davis MT Witte TK Domino JL. (2015). The posttraumatic stress disorder checklist for DSM-5 (PCL-5): Development and initial psychometric evaluation. J. Trauma. Stress. 28:489–98. 10.1002/jts.22059 26606250

[7] Forte G Favieri F Tambelli R Casagrande M. COVID-19 pandemic in the italian population: validation of a post-traumatic stress disorder questionnaire and prevalence of PTSD symptomatology. Int J Environ Res Public Health. (2020) 17:4151. 10.3390/ijerph17114151 32532077 PMC7312976

[8] Liu N Zhang F Wei C Jia Y Shang Z Sun L. Prevalence and predictors of PTSS during COVID-19 outbreak in China hardest-hit areas: gender differences matter. Psychiatry Res. (2020) 287:112921. 10.1016/j.psychres.2020.112921 32240896 PMC7102622

[9] Rodríguez-Rey R Garrido-Hernansaiz H Collado S. Psychological impact and associated factors during the initial stage of the coronavirus (COVID-19) pandemic among the general population in Spain. Front Psychol. (2020) 11:1540. 10.3389/fpsyg.2020.01540 32655463 PMC7325630

[10] Wang C Pan R Wan X Tan Y Xu L Ho CS. Immediate psychological responses and associated factors during the initial stage of the 2019 coronavirus disease (COVID-19) epidemic among the general population in China. Int J Environ Res Public Health. (2020) 17:1729. 10.3390/ijerph17051729 32155789 PMC7084952

[11] Wakeman SE Green TC Rich J. An overdose surge will compound the COVID-19 pandemic if urgent action is not taken. Nat Med. (2020) 26:819–20. 10.1038/s41591-020-0898-0 32555514 PMC8600654

[12] Slavova S Rock P Bush HM Quesinberry D Walsh SL. Signal of Increased Opioid Overdose during COVID-19 from emergency medical services data. Drug Alcohol Depend. (2020) 214:108176. 10.1016/j.drugalcdep.2020.108176 32717504 PMC7351024

[13] Campbell AM An increasing risk of family violence during the Covid-19 pandemic: strengthening community collaborations to save lives. Forensic Sci Int Rep. (2020) 2:100089 10.1016/j.fsir.2020.100089 PMC7152912 38620174

[14] Mazza M Marano G Lai C Janiri L Sani G. Danger in danger: Interpersonal violence during COVID-19 quarantine. Psychiatry Res. (2020) 289:113046. 10.1016/j.psychres.2020.113046 32387794 PMC7190494

[15] Usher K Bhullar N Durkin J Gyamfi N Jackson D. Family violence and COVID-19: Increased vulnerability and reduced options for support. Int J Ment Health Nurs. (2020) 29:549–52. 10.1111/inm.12735 32314526 PMC7264607

[16] Rothwell J The effects of COVID-19 on international labor markets: an update. Brookings. (2020). Available online at: https://www.brookings.edu/research/the-effects-of-covid-19-on-international-labor-markets-an-update/ (accessed July 25, 2020).

[17] Paul KI Moser K Unemployment impairs mental health: Meta-analyses. J Vocat Behav. (2009) 74:264–82. 10.1016/j.jvb.2009.01.001

[18] Maslach C Leiter MP Burnout. In: Rossi AM Perrewe PL editors. SL Sauter Stress and Quality of Working Life: Current Perspectives in Occupational Health. (Information Age Publishing) (2006). p. 42–9.

[19] Maslach C Schaufeli WB Leiter MP. Job burnout. Annu Rev Psychol. (2001) 52:397–422. 10.1146/annurev.psych.52.1.397 11148311

[20] Pines A Aronson E Career Burnout: Causes and Cures. New York, NY: The Free Press (1988).

[21] Di Monte C Monaco S Mariani R Di Trani M From Resilience to Burnout: psychological features of Italian General Practitioners during COVID-19 emergency Provisionally accepted. Front Psychol. (2020) 11:567201 10.3389/fpsyg.2020.567201 33132972 PMC7566043

[22] Cao W Fang Z Hou G Han M Xu X Dong J. The psychological impact of the COVID-19 epidemic on college students in China. Psychiatry Res. (2020) 287:112934. 10.1016/j.psychres.2020.112934 32229390 PMC7102633

[23] Huang Y Zhao N. Generalized anxiety disorder, depressive symptoms and sleep quality during COVID-19 outbreak in China: a web-based cross-sectional survey. Psychiatry Res. (2020) 288:112954. 10.1016/j.psychres.2020.112954 32325383 PMC7152913

[24] Sigdel A Bista A Bhattarai N Poon BC Giri G Marqusee H Depression, Anxiety and Depression-Anxiety Comorbidity Amid COVID-19 Pandemic. (2020). An online survey conducted during lockdown in Nepal. MedRxiv [Preprint]. Available online at: https://www.medrxiv.org/content/10.1101/2020.04.30.20086926v1 (Accessed July **25**:2020). 10.1101/2020.04.30.20086926

[25] Montano RLT Acebes KML COVID stress predicts depression, anxiety and stress symptoms of Filipino respondents. Int J Bus Soc Sci Res. (2020) 9:78–103. 10.20525/ijrbs.v9i4.773

[26] Gaur K Kashri K Sharma A Pachori H (2020). A study of depression, anxiety and insomnia during COVID-19 lockdown in India. Demography India. 49(Special):140–152.

[27] Bäuerle A Teufel M Musche V Weismüller B Kohler H Hetkamp M. Increased generalized anxiety, depression and distress during the COVID-19 pandemic: a cross-sectional study in Germany. J Public Health. (2020) 26:388–95. 10.1093/pubmed/fdaa106 32657323 PMC7454766

[28] Solomou I Constantinidou F. Prevalence and predictors of anxiety and depression symptoms during the COVID-19 pandemic and compliance with precautionary measures: age and sex matter. Int J Environ Res Public Health. (2020) 17:e4924. 10.3390/ijerph17144924 32650522 PMC7400373

[29] Gualano MR Lo Moro G Voglino G Bert F Siliquini R. Effects of Covid-19 lockdown on mental health and sleep disturbances in Italy. Int J Environ Res Public Health. (2020) 17:4779. 10.3390/ijerph17134779 32630821 PMC7369943

[30] Salari N Hosseinian-Far A Jalali R Vaisi-Raygani A Rasoulpoor S Mohammadi M. Prevalence of stress, anxiety, depression among the general population during the COVID-19 pandemic: a systematic review and meta-analysis. Global Health. (2020) 16:57. 10.1186/s12992-020-00589-w 32631403 PMC7338126

[31] Ng QX De Deyn M Lim DY Chan HW Yeo WS. The wounded healer: a narrative review of the mental health effects of the COVID-19 pandemic on healthcare workers. Asian J Psychiatr. (2020) 54:102258. 10.1016/j.ajp.2020.102258 32603985 PMC7305497

[32] Razai MS Oakeshott P Kankam H Galea S Stokes-Lampard H. Mitigating the psychological effects of social isolation during the covid-19 pandemic. BMJ. (2020) 369:m1904. 10.1136/bmj.m1904 32439691

[33] Xiao H Zhang Y Kong D Li S Yang N. The effects of social support on sleep quality of medical staff treating patients with Coronavirus Disease 2019 (COVID-19) in January and February 2020 in China. Med Sci Monit. (2020) 26:e923549. 10.12659/MSM.923921 32132521 PMC7075079

[34] Bruwer B Emsley R Kidd M Lochner C Seedat S. Psychometric properties of the multidimensional scale of perceived social support in Youth. Compr Psychiatr. (2008) 49:195–201. 10.1016/j.comppsych.2007.09.002 18243894

[35] Thoits PA. Social support as coping assistance. J Consult Clin Psychol. (1986) 54:416–23. 10.1037/0022-006X.54.4.416 3745593

[36] Holt-Lunstad J Smith TB Baker M Harris T Stephenson D. Loneliness and social isolation as risk factors for mortality: a meta-analytic review. Perspect Psychol Sci. (2015) 10:227–37. 10.1177/1745691614568352 25910392

[37] Wang Q Hay M Clarke D Menahem S. The prevalence and predictors of anxiety and depression in adolescents with heart disease. J Pediatr. (2012) 161:943–6. 10.1016/j.jpeds.2012.04.010 22640871

[38] Shumaker SC Frazier SK Moser DK Chung ML. Psychometric properties of the multidimensional scale of perceived social support in patients with heart failure. J Nurs Meas. (2017) 25:90–102. 10.1891/1061-3749.25.1.90 28395702

[39] Zhang Y Ma ZF. Impact of the COVID-19 Pandemic on mental health and quality of life among local residents in Liaoning province, China: a cross-sectional study. Int J Environ Res Public Health. (2020) 17:2381. 10.3390/ijerph17072381 32244498 PMC7177660

[40] Chew NW Lee GK Tan BY Jing M Goh Y Ngiam NJ. A multinational, multicentre study on the psychological outcomes and associated physical symptoms amongst healthcare workers during COVID-19 outbreak. Brain Behav Immun. (2020) 88:559–64. 10.1016/j.bbi.2020.04.049 32330593 PMC7172854

[41] Gerhold L (2020). COVID-19: Risk perception and Coping strategies. Results from a survey in Germany. PsyArXiv [Preprint]. Available online at: https://psyarxiv.com/xmpk4/ (Accessed July **20**:2020). 10.31234/osf.io/xmpk4

[42] Chang Y Chan HJ. Optimism and proactive coping in relation to burnout among nurses. J Nurs Manag. (2015) 23:401–8. 10.1111/jonm.12148 24112222

[43] Lall MD Gaeta TJ Chung AS Dehon E Malcolm W Ross A Assessment of physician well-being, part one: burnout and other Negative States. West J Emerg Med. (2019) 20:278–90. 10.5811/westjem.2019.1.39665 30881548 PMC6404708

[44] Fullana MA Hidalgo-Mazzei D Vieta E Radua J. Coping behaviors associated with decreased anxiety and depressive symptoms during the COVID-19 pandemic and lockdown. J Affect Disord. (2020) 275:80–1. 10.1016/j.jad.2020.06.027 32658829 PMC7329680

[45] Shanahan L Steinhoff A Bechtiger L Murray AL Nivette A Hepp U. Emotional distress in young adults during the COVID-19 pandemic: evidence of risk and resilience from a longitudinal cohort study. Psychol Med. (2020) 4:92–100. 10.1017/S003329172000241X 32571438 PMC7338432

[46] Prunas A Sarno I Preti E Madeddu F Perugini M Psychometric properties of the Italian version of the SCL-90-R: A study on a large community sample. Eur Psychiatry. (2012) 27:591–7. 10.1016/j.eurpsy.2010.12.006 21334861

[47] Aben I Verhey F Lousberg R Lodder J Honig A. Validity of the beck depression inventory, hospital anxiety and depression scale, SCL-90, and Hamilton depression rating scale as screening instruments for depression in stroke patients. Psychosomatics. (2002) 43:386–93. 10.1176/appi.psy.43.5.386 12297607

[48] Endler NS Parker JDA. Assessment of multidimensional coping: Task, emotion, and avoidance strategies. Psychol Assess. (1994) 6:50–60. 10.1037/1040-3590.6.1.502348372

[49] Sirigatti S Stefanile C CISS – Coping Inventory for Stressful Situations. Standardizzazione e validazione italiana. Firenze: Giunti O.S. Organizzazioni Speciali (2009).

[50] McWilliams LA Cox BJ Enns MW. Use of the Coping Inventory for Stressful Situations in a clinically depressed sample: factor structure, personality correlates, and prediction of distress. J Clin Psychol. (2003) 59:423–37. 10.1002/jclp.10080 12652635

[51] Zimet GD Dahlem NW Zimet SG Farley GK The multidimensional scale of perceived social support. J Pers Assess. (1988) 52:30–41. 10.1207/s15327752jpa5201_2 2280326

[52] Wittenborn AK Natamba BK Rainey M Zlotnick C Johnson J. Suitability of the Multidimensional Scale of Perceived Social Support as a measure of functional social support among incarcerated adults with major depressive disorder. J Community Psychol. (2020) 48:960–76. 10.1002/jcop.22315 31951288 PMC9365427

[53] Beutel ME Klein EM Brähler E Reiner I Jünger C Michal M. Loneliness in the general population: prevalence, determinants and relations to mental health. BMC Psychiatry. (2017) 17:97. 10.1186/s12888-017-1262-x 28320380 PMC5359916

[54] Domènech-Abella J Mundó J Haro JM Rubio-Valera M. Anxiety, depression, loneliness and social network in the elderly: longitudinal associations from The Irish Longitudinal Study on Ageing (TILDA). J Affect Disord. (2019) 246:82–88. 10.1016/j.jad.2018.12.043 30578950

[55] Erzen E Çikrikci Ö. The effect of loneliness on depression: a meta-analysis. Int J Soc Psychiatr. (2018) 64:427–35. 10.1177/0020764018776349 29792097

[56] Hedley D Uljarevi ć M Foley KR Richdale A Trollor J. Risk and protective factors underlying depression and suicidal ideation in Autism Spectrum disorder. Depress Anxiety. (2018) 35:648–57. 10.1002/da.22759 29659141

[57] Stickley A Koyanagi A. Loneliness, common mental disorders and suicidal behavior: Findings from a general population survey. J Affect Disord. (2016) 197:81–7. 10.1016/j.jad.2016.02.054 26971125

[58] Teo AR Marsh HE Forsberg CW Nicolaidis C Chen JI Newsom J. Loneliness is closely associated with depression outcomes and suicidal ideation among military veterans in primary care. J Affect Disord. (2018) 230:42–9. 10.1016/j.jad.2018.01.003 29407537

[59] van Winkel M Wichers M Collip D Jacobs N Derom C Thiery E. Unraveling the role of loneliness in depression: the relationship between daily life experience and behavior. Psychiatry. (2017) 80:104–17. 10.1080/00332747.2016.1256143 28767331

[60] Long H Fu MX Hai RL. Emotional Responses and Coping Strategies of Nurses and Nursing College Students During COVID-19 Outbreak. (2020). Available online at: https://www.medrxiv.org/content/10.1101/2020.03.05.20031898v1 (Accessed by July 25, 2020).

[61] McClelland H Evans JJ Nowland R Ferguson E O'Connor RC. Loneliness as a predictor of suicidal ideation and behaviour: a systematic review and meta-analysis of prospective studies. J Affect Disord. (2020) 274:880–96. 10.1016/j.jad.2020.05.004 32664029

[62] Peplau LA Perlman D Perspectives on loneliness. In: Peplau LA editor. Loneliness: A Sourcebook of Current Theory, Research and Therapy. Wiley (1982). p. 1–8.

[63] Bäuerle A Skoda EM Dörrie N Böttcher J Teufel M. Psychological support in times of COVID-19: the Essen community-based CoPE concept. J Public Health. (2020) 42:649–50. 10.1093/pubmed/fdaa053 32307516 PMC7188143

[64] Mariani R Di Trani M Negri A Tambelli R. Linguistic Analysis of Autobiographical Narratives in Unipolar and Bipolar Mood Disorders in Light of Multiple Code Theory. J Affect Disord. (2020) 273:24–31. 10.1016/j.jad.2020.03.170 32421609

